# Coping as a mediator between family stressors and behavioural adjustment in siblings of children with progressive life-limiting conditions: a multilevel longitudinal study

**DOI:** 10.1186/s12887-026-07110-z

**Published:** 2026-06-08

**Authors:** Joanne Tay, Kimberley Widger, Robyn Stremler, Jason D. Pole

**Affiliations:** 1 Faculty of Nursing, University of Windsor, Room 336, Toldo Health Education Centre, 401 Sunset Avenue, Windsor, ON N9B 3P4 Canada; 2 Lawrence S. Bloomberg Faculty of Nursing, University of Toronto, Toronto, Canada; 3 Paediatric Advanced Care Team, Hospital for Sick Children, Toronto, Toronto, Canada; 4 Life Stage Program, ICES, Toronto, Toronto, Canada; 5 Child Health Evaluative Sciences, Hospital for Sick Children, Toronto, Canada; 6 Centre for Health Services Research, The University of Queensland, Brisbane, Australia; 7 Cancer Research Program, ICES, Toronto, Canada; 8 Dalla Lana School of Public Health, University of Toronto, Toronto, Canada

**Keywords:** Siblings, Progressive life-limiting conditions, Coping strategies, Multilevel mediation, Behavioural adjustment

## Abstract

**Background:**

Siblings of children with progressive life-limiting conditions face elevated emotional and behavioural risks. Although multiple family stressors are known, how cumulative stress shapes sibling adjustment is unclear. This study examined how illness-related family stressors affect sibling behavioural outcomes and whether active versus passive coping strategies are associated with these relationships.

**Methods:**

We conducted secondary analysis of longitudinal data from the Charting the Territory (CTT) study, examining 70 siblings (aged 7–18 years) from 58 families with a child diagnosed with progressive life-limiting conditions. Families were categorized into two treatment-duration groups based on time since first seeking medical treatment for the ill child (≤ 1 year vs. > 1 year). Using multilevel exploratory factor analysis, we empirically derived a latent "Siblings' Stressor" construct from nine potential variables. Multilevel mediation models examined relationships between Siblings' Stressor, coping strategies (Kidcope), and three behavioural outcomes (Child Behaviour Checklist): internalizing, externalizing, and total behaviour problems across baseline (defined as each family's first assessment wave at study enrollment), 6-month, and 12-month assessments.

**Results:**

The Siblings' Stressor construct comprised parental stress, anxiety, depression, and caregiving burden. Significant direct effects emerged between Siblings' Stressor and all behavioural outcomes. Coping associations differed by treatment-duration group. For internalizing problems, active coping provided consistent protection across early (≤ 1 year: -.052, 95% CI [-.103, -.001]) and longer-term families (> 1 year: -.109, 95% CI [-.121, -.097]), while passive coping demonstrated risk effects. However, for externalizing and total behaviour problems, active coping showed different associations by treatment-duration group, being protective in early-treatment families (≤ 1 year) and positively associated with externalizing (.048, 95% CI [.021, .075]) and total behaviour problems (095, 95% CI [.053, .136]) in longer-treatment families (> 1 year). Passive coping was linked to elevated externalizing and total behaviour problems in both groups, with particularly strong associations in families within their first year of treatment.

**Conclusion:**

Parental psychological distress and caregiving burden were associated with sibling adjustment across all behavioural outcomes, with coping strategies showing differential patterns by treatment-duration group. Active coping was related to fewer internalizing difficulties, while passive coping was associated with elevated risk. These findings support family-centred approaches that address parental mental health and promote developmentally appropriate coping strategies across the illness trajectory.

**Supplementary Information:**

The online version contains supplementary material available at 10.1186/s12887-026-07110-z.

## Background

Children living with progressive, life-limiting conditions – referred to as Quadrant 3 (Q3) conditions [3, 49] – such as Batten disease, Duchenne muscular dystrophy, and mitochondrial disorders experience complex and ongoing medical needs that have wide-reaching effects on the family unit [5, 19]. Healthy siblings, in particular, often face emotional and behavioural challenges linked to this prolonged caregiving environment. Studies have identified elevated risks for psychosocial difficulties in siblings, including compromised emotional functioning [52], behavioural problems [4, 33], academic struggles [20], and strained family relationships [60]. While some siblings adapt well [34], others experience emotional distress or engage in risky behaviours during adolescence [23, 28]. These issues often manifest as internalizing (e.g., anxiety, depression) or externalizing (e.g., aggression, defiance) symptoms [12].

The origins of these difficulties are multifactorial, arising from the child's illness characteristics, family dynamics, and limited parental resources available to healthy siblings [24, 43]. Yet prior research has tended to examine these factors separately, overlooking their cumulative effects. Sameroff [45] highlighted the importance of considering the combined influence of ecological risks. Building on this perspective, Long et al. [32] proposed a "contextual threat" model that integrates illness-related, social, and psychological stressors to explain sibling adjustment. Long et al.'s model conceptualizes threat as emerging from disruptions in the family system, changes in parental availability, emotional climate, and daily routines that shape children's appraisals of safety, predictability, and control.

Despite this conceptual advancement, empirical findings linking stress exposure to sibling outcomes remain inconsistent. Some studies report associations between family dysfunction and poorer sibling mental health [24, 26], whereas others do not [7]. Similarly, findings regarding parental distress and caregiving strain [15, 29, 30], as well as illness severity [43], vary across studies. One explanation for these inconsistencies is that contextual threat domains have rarely been operationalized collectively or tested as a cumulative construct. As a result, it remains unclear how multiple stress inputs interact to shape sibling adjustment. A second explanation is that prior work has insufficiently examined siblings' active responses to stress.

To address this variability, we draw on Lazarus and Folkman's [31] Transactional Stress and Coping Model, which posits that psychological outcomes depend not only on stress exposure but also on how individuals appraise and respond to stressors. Research grounded in this model demonstrates that coping strategies differentially influence adjustment. Passive coping strategies, such as avoidance, withdrawal, or wishful thinking, are generally associated with higher levels of anxiety and depression [14]. In contrast, active coping strategies, including problem-solving, support-seeking, and emotion regulation, are associated with more adaptive outcomes [44]. Coping effectiveness, however, depends on stressor controllability, chronicity, and developmental context [12, 14, 16],Zimmer-Gembeck & Skinner, 2016). Siblings may rely on maladaptive coping patterns such as avoidance or withdrawal [55], which can contribute to emotional and behavioural difficulties [27]. Coping may therefore serve as a key mechanism linking cumulative stress exposure to sibling adjustment.

Although coping processes have been examined among siblings of children with cancer [23, 24, 57], less is known about siblings of children with Q3 conditions, which are characterized by prolonged and uncertain trajectories that often span many years and impose sustained emotional and logistical demands on families, potentially intensifying cumulative stress exposure.

Building on Long et al.'s [32] contextual threat model, the present study integrates illness-related, parental, and family-level stressors into a cumulative construct, incorporates coping as a mediating process, and examines how demographic and contextual factors shape these relationships.

### Aim

This study aimed to advance understanding of the psychosocial adjustment of siblings of children with progressive life-limiting conditions by examining three interrelated objectives:

1. To assess how illness-related stressors influence sibling emotional and behavioural outcomes, specifically internalizing, externalizing, and total behaviour problems.
2. To examine how variations in coping strategies, particularly active versus passive, mediate the effects of these stressors on psychosocial adjustment.
3. To explore how selected demographic and contextual factors (e.g., time since seeking treatment for the ill child, gender, and birth order) shape these relationships.

We hypothesized that:

- In alignment with Objective 1, siblings' illness-related stressors will be positively associated with internalizing, externalizing, and total behaviour problems.
- As relevant to both Objectives 1 and 2, active coping strategies will mediate the relationship between illness-related stressors and siblings' emotional and behavioural problems, reducing the negative impact of these stressors.
- Also aligned with Objectives 1 and 2, we expected passive coping strategies to mediate the relationship between illness-related stressors and siblings' emotional and behavioural problems and, on average, to be associated with higher levels of these problems, consistent with prior work linking avoidance- and withdrawal-based coping to poorer adjustment.
- Addressing Objective 2 specifically, we expected active coping strategies to show more favourable associations with psychosocial adjustment than passive coping, with active coping linked to lower levels of anxiety, depression, and behavioural issues on average, while recognizing that the adaptiveness of specific strategies may vary by context.
- In relation to Objective 3, we explored whether demographic and contextual factors (e.g., gender, birth order, and time since the ill child first sought treatment) moderate the relationships among stressors, coping processes, and psychosocial outcomes, given prior evidence that these factors meaningfully shape siblings’ exposure to family stress and opportunities for coping, and whether associations of active and passive coping with internalizing, externalizing, and total behaviour problems differed between families whose child had been in treatment for ≤ 1 year versus > 1 year.

## Methods

### Study overview

The present study developed an integrated approach tailored to the experiences of siblings of children with Q3 conditions. We empirically operationalized the contextual threat construct by identifying the domains most relevant to siblings and deriving a composite variable, "Siblings' Stressor," to capture the cumulative burden of family, parental, and child-level stressors. Specifically, we initially selected nine contributing variables (Family Adaptation and Cohesion, Parental Stress, Parental Anxiety, Parental Depression, Parental Grief, Burden of Caregiving, Marital Satisfaction, and Symptom Severity of the Child with Q3 Condition) that corresponded to Long et al.'s categories and were assessed in the Charting the Territory (CTT) dataset. Through multilevel exploratory factor analysis, we empirically derived which of these variables best represented the underlying stressor construct. We also incorporated coping mechanisms as mediating processes to test whether active and passive coping explain the relationship between cumulative stress and sibling outcomes. Finally, we examined how demographic and contextual factors such as sibling birth order, gender, and the time since the family first sought treatment might influence these stress and coping experiences, given that these factors meaningfully structure siblings' exposure to caregiving responsibilities, access to parental attention, and the duration and intensity of illness-related demands known to affect children's stress appraisals and coping patterns.

### Study design and data source

This study is a secondary analysis of data from the CTT study [46], a longitudinal cohort study that followed children aged < 19 years with Q3 conditions and their families to collect data at regular intervals about the biopsychosocial experiences of the ill child, parents, and siblings. Recruitment took place between July 2009 and October 2012 via convenience sampling at nine clinical sites across Canada and the United States. Most families were identified through pediatric hospital clinics (49%) and palliative care services (43%). Families qualified for the broader CTT study if they had a child with a medical condition characterized by five key features: (1) physician confirmation of a progressive, non-curable diagnosis, (2) suspected genetic or metabolic etiology, (3) presence of central nervous system dysfunction, (4) absence of effective curative treatments or treatment failure in the specific case, and (5) anticipated life expectancy not extending beyond 20 years of age. The present study received research ethics approval from the University of Toronto.

### Participants

The CTT cohort included 258 families, of which 385 were initially evaluated for eligibility. Of those not enrolled, 34 did not meet the inclusion criteria, 59 declined participation, and 34 could not be contacted. This secondary analysis comprises only those families with > 1 biological, adoptive, or step-sibling of the ill child who were 7–18 years of age, had no cognitive impairment and/or severe health conditions, and consented to participate. Some families included more than one sibling; thus, 70 siblings in 58 families were eligible for inclusion in the analysis. The siblings ranged in age from 7 to 18 years (M = 11.2, SD = 3.2), providing coverage across late childhood and adolescence. Of the 58 families, 48 had one participating sibling, 6 had two, and 4 had three. A full participant flow diagram for the original cohort has been published elsewhere [50] and provides a detailed overview of recruitment and enrollment procedures for the CTT study.

In each family, the designated primary caregiving parent completed the parent questionnaires. This referred to the parent identified by the family as taking primary responsibility for the care and coordination of the ill child's medical and day-to-day needs, who was also assumed to have the broadest overview of the family's overall functioning, including the healthy sibling's wellbeing. In families where caregiving responsibilities were divided between parents, only the primary designated caregiver participated. The majority of respondents were mothers, with only four fathers serving as the designated parent respondent in this subsample. The same designated parent completed all parent-report questionnaires for that family. Given the small number of father respondents, parent-report data were treated as from a single primary caregiver rather than analyzed separately by mother or father.

### Procedure

Data collection involved multiple questionnaire components administered to different family members: (1) demographic information collected at baseline (i.e., first assessment at study enrollment) with selective updates every six months, (2) monthly assessments of the ill child's health status and functional capacity, and (3) six-monthly evaluations of family psychosocial well-being and sibling behavioural patterns. Healthy siblings aged 7–18 also completed coping assessments every six months.

The initial data collection session was conducted through face-to-face meetings (*n* = 24) or postal surveys (*n* = 34), with all subsequent assessments administered via mail every six months. To ensure participant privacy, each family member received an individual response envelope, which was then consolidated into a single-family packet for return to the research team. In cases where the ill child passed away during the study period (*n* = 8), families received a bereavement notification and were given a six-month respite from data collection before being recontacted about continued participation. Following previous publications using this dataset (Tay, Widger, Steele, et al., 2020), our analysis utilized data from the initial three assessment waves (baseline, 6-month follow-up, and 12-month follow-up) due to sample attrition in later waves. This timeframe provided adequate opportunity to detect meaningful changes in psychosocial outcomes and coping patterns, given the six-month intervals between assessments recommended for detecting change in these domains [6]. The mean duration between consecutive assessment points was 6.15 months (SD = 2.30).

### Variables and measures

#### Independent variables

##### Siblings' stressor

Based on Lazarus and Folkman's [31] stress and coping framework, we initially identified nine potential contextual stressor variables: family adaptation, family cohesion, parental stress, anxiety, depression, caregiving burden, marital satisfaction, grief, and the ill child's symptom severity. Rather than creating a simple composite by averaging these variables, we employed multilevel exploratory factor analysis (EFA) to empirically derive a latent Siblings' Stressor construct that accounted for the nested structure of siblings within families. This data-driven approach allowed us to identify which observed variables best represented the underlying stressor construct at both individual and family levels across the three time points, consistent with best practices in scale development and validation.

The following measures served as indicators of the Siblings' Stressor construct.

##### Family adaptation and cohesion

Parents completed the Family Adaptation and Cohesion Evaluation Scale (FACES III; [41], a 20-item scale rated on a 5-point scale. Mean scores for the adaptation and cohesion subscales were computed separately by averaging the scores of all 10 questions in each subscale. FACES III has demonstrated good validity and reliability [24], with Cronbach's alpha of 0.70 for adaptation and 0.83 for cohesion in the current sample.

##### Parental stress

Parents' perceived stress was measured using the Perceived Stress Scale (PSS), which comprises 10 items rated on a 5-point scale [10]. The PSS demonstrated good internal consistency in the current sample (α = 0.89) and has shown good reliability and validity in previous research [40]. The mean score for perceived stress for each parent was computed.

##### Parental anxiety

Parents completed the State-Trait Anxiety Inventory (STAI) state form, which comprises 20 items rated on a 4-point scale [47]. The mean anxiety score for each parent was computed. The STAI has shown good reliability and validity [51], with excellent internal consistency observed in the current sample (α = 0.94).

##### Parental depression

Parents' level of depressive symptoms was measured using the Center for Epidemiologic Studies Depression Scale (CES-D; [42]. This 20-item questionnaire uses a 4-point scale, with excellent internal consistency in the current sample (α = 0.91), and a mean score was computed for each parent. The CES-D has shown good reliability and validity [35].

##### Parental grief

Parental grief was measured using the Grief Scale, adapted from the Inventory of Social Support Scale [56]. This 6-item questionnaire consists of one item rated on a 10-point scale and five items rated on a 5-point scale. The scale showed good internal consistency in the current sample (α = 0.83), and the mean score for grief was computed for each parent.

##### Burden of caregiving

The parents' level of caregiving burden was assessed using the Burden Scale [37], which consists of 4 items rated on a 4-point scale. The Burden Scale demonstrated good internal consistency in the current sample (α = 0.82) and has shown good reliability and validity in previous research [9]. The mean score was computed.

##### Marital satisfaction

Parents reported their marital satisfaction using Norton's Quality Marriage Index (QMI; [39], a 6-item scale with 5 items rated on a 7-point Likert scale and an overall rating of happiness on a 10-point Likert scale. An overall mean score was calculated. The QMI has shown good reliability and validity [8], with excellent internal consistency observed in the current sample (α = 0.96).

All parent-report variables above were conceptualized as indicators of the family's overall psychosocial state and contextual threat, rather than as mother- or father-specific characteristics. Because only one designated primary caregiving parent per family completed the questionnaires, and very few respondents were fathers, we did not have sufficient power to model mothers and fathers as distinct groups in this analysis.

##### Symptom severity of the child with Q3 condition

Parents completed the Clinical Symptom Checklist, created by the CTT study team and based on two existing tools: the Pediatric Quality of Life and Evaluation of Symptoms Technology (PediQUEST; [58] and the revised Memorial Symptom Assessment Scale (7–12 years, [11]. Parents completed the checklist every month,however, for analysis we used only data collected at timepoints corresponding with the biopsychosocial data collection (i.e., baseline, 6 months, and 12 months). As previous research indicates that healthy siblings are most affected by easily visible physical symptoms [59], we used only responses to items on pain, difficulty breathing, alertness, and seizures. Parents reported on the frequency of symptoms, changes since the previous assessment, and the level of distress. The total score ranges from 0 to 28, with higher scores indicating greater symptom severity.

#### Dependent variables

##### Child emotional and behavioural problems

Parents completed the Child Behaviour Checklist (CBCL; [2] for each participating sibling. The CBCL comprises 113 questions rated on a 3-point Likert scale. Items are combined into three composite scales, creating three separate dependent variables: (1) Internalizing Problems, reflecting primarily inwardly directed emotional symptoms (e.g., anxiety, depression, somatic complaints),(2) Externalizing Problems, reflecting outwardly directed behavioural symptoms (e.g., aggression, rule-breaking); and (3) Total Behaviour Problems, a broader index of overall psychosocial difficulties calculated as the sum of all CBCL problem items across syndrome scales, incorporating internalizing and externalizing difficulties as well as additional domains (e.g., attention, social, and thought problems) not fully captured by the two specific composites. These three outcomes allow examination of how stressors and coping strategies differentially impact emotional problems, behavioural problems, and overall psychosocial adjustment. Reliability and validity of the CBCL composite scales have been documented [24], and Stephenson et al. [50] reported good internal consistency for the current sample. In the current study, Cronbach's alpha was 0.88 for Internalizing Problems, 0.93 for Externalizing Problems, and 0.96 for Total Behaviour Problems.

#### Mediator variable

##### Use of coping strategies frequency

In the present study, coping strategies were categorized into two broad composites: active coping and passive coping. Consistent with control-based models of coping [12, 14], active coping was defined as efforts to engage with the stressor or regulate one's emotional response, including strategies such as problem-solving, seeking social support, and cognitive reappraisal. Passive coping was defined as disengagement-oriented efforts involving avoidance, withdrawal, denial, or wishful thinking, reflecting attempts to distance oneself from the stressor rather than directly addressing it. Active and passive coping were treated as broad heuristic composites rather than discrete categories, reflecting the aggregated structure of the Kidcope subscales. Composite scores were created by aggregating relevant subscales from the Kidcope instrument, with higher scores indicating greater use of that coping style.

Siblings' use of coping strategies was measured using two versions of Kidcope [48]. Siblings aged 7 to 12 years completed the younger version (*n* = 40), whereas those aged 13 to 18 completed the older version (*n* = 30). Kidcope measures 10 coping strategies that can be combined into the two higher-order composites described above. In the present study, the active coping composite collapsed across several Kidcope strategies (e.g., problem-focused efforts, positive thinking, and support seeking), so we did not examine the effects of individual active strategies separately. The scale demonstrated good to excellent internal consistency in the current sample across both versions: active coping α = 0.80 (older) and α = 0.83 (younger),passive coping α = 0.82 (older) and α = 0.88 (younger). Although siblings were asked to rate both the frequency and efficacy of coping strategies, we used only frequency ratings, as our focus was on examining coping strategy use patterns. Item scores were averaged and converted to standardized T-scores, allowing scores from the younger and older Kidcope versions to be combined in analysis [16]. Kidcope has shown good reliability and validity with a similar population [21].

### Covariates

We initially considered key demographics as potential covariates: household income, parents' education level, siblings' gender, birth order (i.e., older vs. younger than the ill child), and age at study enrollment. Given the challenges of confirming a diagnosis in some children with Q3 conditions [53], we created a "time since first sought treatment" variable based on the difference between the date parents reported that they first sought medical treatment for their child and the date of study enrollment. This variable was dichotomized into ≤ 1 year versus > 1 year to distinguish families in the early phase of illness management from those in a more prolonged phase, consistent with literature suggesting that family stress and psychosocial adaptation processes may shift following diagnosis or treatment initiation [17, 38]. Final covariate inclusion was determined based on bivariate correlations with outcome variables.

### Data analysis

#### Preliminary analyses

Data from families in which the ill child died (n = 8) were included only up to the assessment immediately preceding bereavement; post-bereavement data were excluded. Additional families withdrew or missed later assessments for reasons unrelated to bereavement. All available observations were retained, and missingness arising from attrition or bereavement-related pauses was handled using full-information maximum likelihood (FIML), consistent with recommended approaches for longitudinal multilevel SEM. Initial analyses involved examining descriptive statistics for all study variables, including means and standard deviations for continuous measures and frequencies and percentages for categorical and dichotomous variables. We also compared the ≤ 1-year and > 1-year treatment-duration groups on key demographic and illness-related variables (sibling age, gender, birth order, parent education, household income, and child symptom severity) using t-tests and χ^2^ tests to assess potential differences between groups. Pearson product-moment correlations were used to assess relationships among continuous variables of interest. Point-biserial correlations were used for associations between continuous and dichotomous variables (i.e., gender, birth order). Both were used to identify relevant covariates for inclusion in multilevel models. The alpha level for statistical significance was set at p < 0.05.

#### Multilevel factor analysis for siblings’ stressor

Given the nested structure of the data, with multiple siblings nested within the same families (*n* = 21 children from 9 families), we conducted a multilevel exploratory factor analysis (EFA) in Mplus 8.4 to empirically derive a latent construct representing siblings' stressors. This approach modelled the factor structure separately at the within-family (Level 1) and between-family (Level 2) levels across all three time points, accounting for the non-independence of observations due to family clustering [36]. However, due to the small number of clusters (*n* = 9 families), Level 2 factor estimates should be interpreted with caution, as small sample sizes at this level may limit the stability and reliability of parameter estimation. Indicators with factor loadings < 0.40 or inconsistent loadings across time points and levels were excluded from the final construct. Model fit was evaluated using established criteria: RMSEA < 0.08, CFI and TLI ≥ 0.90, and SRMR < 0.08.

#### Multilevel mediation models

After identifying the Siblings' Stressor factor, we estimated multilevel latent growth curve models in Mplus 8.4 to examine mediation effects. Although three time points were collected, preliminary analyses confirmed prior findings [53] that significant variance existed primarily in intercepts rather than in growth trajectories for the outcome variables. Accordingly, multilevel mediation models focused on initial levels (intercepts) of all constructs rather than change over time. Time since first sought treatment was modelled as a Level 2 grouping variable (≤ 1 year vs. > 1 year), and mediation effects are therefore interpreted as differences between families with shorter versus longer treatment durations rather than intra-individual change over time.

Three separate models examined the indirect effects of the initial level of Siblings' Stressor on the initial levels of each behavioural outcome (internalizing, externalizing, and total behaviour problems) through the initial levels of active and passive coping at both Level 1 and Level 2. Only covariates showing significant bivariate correlations with outcome variables were included as Level 2 predictors to improve model parsimony and statistical power. For each model, total, direct, and indirect effects were estimated at both levels. The direct effect represented the association between the initial level of Siblings' Stressor and the initial level of the respective outcome, controlling for the initial levels of active and passive coping. Specific indirect effects through each coping pathway were also examined at both levels. To ensure robustness of parameter estimates, bias-corrected bootstrap confidence intervals (95%) were computed for all indirect effects. Model fit was evaluated using RMSEA < 0.08, CFI/TLI ≥ 0.90, and SRMR < 0.08. FIML estimation was employed to address missing data at both levels of the model.

## Results

Data from 70 siblings in 58 families were analyzed. The children with Q3 conditions represented a heterogeneous group of rare, life-limiting diagnoses. In the broader CTT cohort from which this sibling subsample was drawn (275 index children), the most common diagnostic categories were multiorgan congenital abnormalities (*n* = 54, 19.6%), severe neurological impairment not yet diagnosed (*n* = 45, 16.4%), lysosomal/peroxisomal leukodystrophies (*n* = 44, 16.0%), mitochondrial encephalopathy/myopathy (*n* = 31, 11.3%), and other neurodegenerative diseases (*n* = 23, 8.4%), with less frequent diagnoses including structural central nervous system abnormalities (8.0%), epileptic encephalopathies (6.9%), small molecule diseases (4.7%), neuromuscular diseases (3.6%), other metabolic diseases (1.1%), congenital disorders of glycosylation (2.5%), and other conditions not otherwise specified (1.5%). Participating siblings ranged in age from 7 to 18 years at enrollment (M = 11.31, SD = 3.23), with 55.7% being female. Most siblings (65.7%) were older than their siblings with the Q3 condition, indicating that in the majority of families the sibling had at least some developmental advantage in age. Families were categorized into two treatment-duration groups based on time since first sought treatment: ≤ 1 year (n = 12 siblings) and > 1 year (n = 58 siblings). The ≤ 1 year and > 1 year groups did not differ significantly in sibling age, gender, birth order, parental education, household income, or child symptom severity (all p-values > 0.10), suggesting that treatment-duration effects are unlikely to be explained by these demographic differences. Complete data for the multilevel analyses were available for all 70 siblings across the three time points used in this study. Parent-reported behavioural assessments (CBCL) were completed for all participating siblings, while sibling-reported measures were completed by those aged 11–18 years; 42 siblings (60.0% of the sample) were in this age range and thus eligible to complete the YSR and Kidcope at one or more time points, resulting in a subset of participants for self-report analyses. Parental and sibling demographic characteristics, as well as summary scores for each study variable, are reported in Table 1.

**Table 1:** Descriptive information of parents and siblings

**Variables**	
Parents (*n* = 58)	
Parents’ age in years, Mean (SD)	39.5(7.1)
Marital status	n (%)
Married/living as married	51 (87.9)
Divorced/separated/not presently married/widowed	7 (12.1)
Parents’ education level	n (%)
High school diploma or less	12 (20.7)
At least some college or university education	46 (79.3)
Average household income	n (%)
< $40,000	13 (23.2)
$40,000—< $80,000	20 (35.7)
$80,000—$120,000	16 (28.6)
> $120,000	7 (12.5)
Summary scores for study variables reported by parents (possible range; sample range)	Mean (SD)
FACES III – Family adaptation (10–50; 15.0–29.0)	22.7 (5.8)
FACES III – Family cohesion (10–50; 27.0–50.0)	40.2 (4.6)
PSS – Parental stress (0–40; 3.0–30.0)	19.5 (5.9)
STAI – Parental anxiety (20–80; 23.0–59.0)	42.7 (11.3)
CES-D – Parental depression (0–60; 4.7–42.0)	14.9 (9.7)
Grief Scale – Parental grief (6–35; 7.7–30)	24.2 (3.6)
Burden Scale – Burden of caregiving (4–16; 5.3–16.0)	11.6 (3.2)
QMI – Marital satisfaction (6–45; 16.0–45.0)	33.0 (3.4)
Clinical Symptom Checklist – Ill child’s symptom severity (0–28; 3.2–15.0)	7.5 (5.2)
Siblings (*n* = 70)	
Siblings’ age in years, Mean (SD)	11.2 (3.2)
Time since first sought treatment	n (%)
≤ 1 year	12 (17.1)
> 1 year	58 (82.9)
Siblings’ gender	n (%)
Male	31 (44.3)
Female	39 (55.7)
Siblings’ birth order	n (%)
Older relative to the ill child	46 (65.7)
Younger relative to the ill child	24 (34.3)
Summary scores of siblings’ problems and coping reported by siblings (possible range; sample range)	Mean (SD)
CBCL – Internalizing problems (0–64; 3.0–24.7)	7.2 (5.8)
CBCL – Externalizing problems (0–70; 3.0–32.7)	6.8 (6.6)
CBCL – Total behaviour problems (0–226; 3.4–88.3)	25.1 (18.4)
Kidcope – Active Coping (0–100)	50 (4.2)
Kidcope – Passive Coping (0–100)	61.3 (6.8)

*CBCL* Child Behaviour Checklist, *FACES III* Faces Adaption and Cohesion Scale III, *CES-D* Center for Epidemiologic Studies Depression Scale, *STAI* State-Trait Anxiety Inventory, *PSS* Perceived Stress Scale, *QMI* Quality of Marriage Index. Means and SDs are presented for the full sample, 58 parents, and 70 siblings

### Multilevel exploratory factor analysis for siblings’ stressor

The multilevel EFA revealed that three of the nine initially proposed variables (family adaptation, parental grief, and marital satisfaction) did not demonstrate adequate factor loadings (> 0.40) or consistent loadings across time points and levels and were therefore excluded from the derived latent construct. The analysis initially suggested a two-factor solution, with the first factor comprising four observed variables (parental stress, parental anxiety, parental depression, and parental caregiving burden), and the second comprising two observed variables (family cohesion and the child's symptom severity in the Q3 condition). However, only the first factor demonstrated stability and met our a priori criteria for factor retention across all three time points at both the within-family (Level 1) and between-family (Level 2) levels. Since only this first factor demonstrated consistent stability, it was retained as the Siblings’ Stressor latent construct for subsequent multilevel mediation analyses, representing parental psychological distress and caregiving burden as the core stressor construct. The factor loadings for the retained indicators were strong and consistent across the three time points: Parental stress (0.852, 0.937, 0.934), parental anxiety (0.854, 0.842, 0.961), parental depression (0.842, 0.860, 0.897), and parental burden of caregiving (0.602, 0.857, 0.745). Standardized factor loadings for the Siblings’ Stressor latent variable at each time point are presented in Supplementary Table S1. Multilevel model fit indices indicated excellent fit to the data (RMSEA = 0.03, CFI = 0.99, TLI = 0.98, SRMR-within = 0.02, SRMR-between = 0.01), supporting the validity of the derived latent construct of Siblings’ Stressor within the multilevel framework (see Table 2).

**Table 2:** Model fit indices for multilevel structural equation models

Model	χ^2^	RMSEA	CFI	TLI	SRMR-Within	SRMR- Between	ICC
EFA Model							
Siblings’ Stressor	.294 (2)	.03	.99	.98	.02	.01	.18
Mediation Models							
Internalizing Problems	136.43 (93)	.05	.98	.98	.03	.02	.24
Externalizing Problems	125.61 (79)	.01	.96	.98	.02	.01	.31
Total Behaviour Problems	132.93 (115)	.02	.99	.99	.03	.02	.28

*χ*^*2*^ chi-square, *RMSEA* Root Mean Square Error of Approximation, *CFI* Bentler’s Comparative Fit Index, *TLI* Tucker-Lewis Index, *SRMR* Standardized Root Mean Square Residual, *ICC* Intraclass Correlations. All models used TYPE = TWOLEVEL with families as clustering units (*N* = 70 siblings, 58 families)

### Multilevel correlation analysis

#### Covariate selection

Table 3 presents the bivariate correlations among the initial levels of the study variables. The initial level of Siblings' Stressor and the initial levels of active and passive coping were significantly correlated with the initial levels of internalizing, externalizing, and total behaviour problems. In contrast, siblings' gender, birth order, and parents' education level were not significantly associated with siblings’ stressor, coping strategies, or behavioural outcomes (all p > 0.05). Consequently, these variables were excluded from subsequent multilevel mediation models to improve model parsimony and statistical power. Covariates demonstrating significant correlations with the outcome variables (time since first seeking treatment, siblings' age, and household income) were retained as Level 2 predictors in the multilevel mediation models.

**Table 3:** Bivariate correlations of study variables (*N* = 70)

	1	2	3	4	5	6	7	8	9	10	11	12
Covariates												
1. Gender	-											
2. Birth order	-.177	-										
3. Age	.220	.670	-									
4. Time since first sought treatment	.273*	.913	-.670*	-								
5. Education level	.106	.250	.500	.621	-							
6. Household income	-.180	.720	.913	.864*	.520	-						
Independent variable												
7. Sibling stressors	-.190	.360	.871*	.663*	.233	.425*	-					
Mediating variables												
8. Active coping	.313	.415	.633*	.821*	-.365	.563	.830*	-				
9. Passive coping	.639	.332	-.529*	-.811*	-.284	.761	.828*	.126*	-			
Dependent variables												
10. Internalizing problems	.875	.232	.689*	.861*	-.211	-.367	.982**	-.880*	.910*	-		
11. Externalizing problems	.549	.344	-.548*	-.662*	-.349	-.226	.730*	-.828*	.952*	.987*	-	
12. Total behaviour problems	.461	.525	-.699*	-.784*	-.410	-.488	.659*	-.886*	.930*	.925*	.703*	-

Pearson product-moment correlations were used for continuous variable pairs. Point-biserial correlations were used for associations involving dichotomous variables (gender, birth order)

^*^p <.05

^**^p <.01

### Multilevel mediation models

#### Model specification, fit, and variance components

Three primary multilevel mediation models were examined to investigate the indirect effects of the initial level of Siblings' Stressor on the initial levels of internalizing, externalizing, and total behaviour problems, mediated by the initial levels of active and passive coping, while accounting for the nested family structure. The overall fit indices for all three multilevel mediation models indicated good to excellent model fit (for the internalizing model: RMSEA = 0.05, CFI = 0.98, TLI = 0.98, SRMR-Within = 0.02, SRMR-Between = 0.03; for the externalizing model: RMSEA = 0.01, CFI = 0.96, TLI = 0.98, SRMR-Within = 0.01, SRMR-Between = 0.02; and for the total behavior problems model: RMSEA = 0.02, CFI = 0.99, TLI = 0.99, SRMR-Within = 0.02, SRMR-Between = 0.03), suggesting that the models adequately accounted for the variance at both the individual and family levels. Intraclass correlations indicated moderate clustering within families for all variables (ICCs ranged from 0.15 to 0.35), confirming the appropriateness of the multilevel approach. Significant variance was observed at the individual level (Level 1) for the intercepts of Siblings' Stressor (Var = 27.12, *p* = 0.023), active coping (Var = 35.67, p = 0.009), passive coping (Var = 18.66, p = 0.003), internalizing problems (Var = 21.93, p = 0.012), externalizing problems (Var = 24.86, p = 0.007), and total behaviour problems (Var = 31.56, p = 0.003), indicating meaningful variation across individuals in their initial levels of these constructs and justifying the multilevel analytical approach.

#### Multilevel mediation models for internalizing, externalizing, and total behaviour problems

**Fig. 1:**
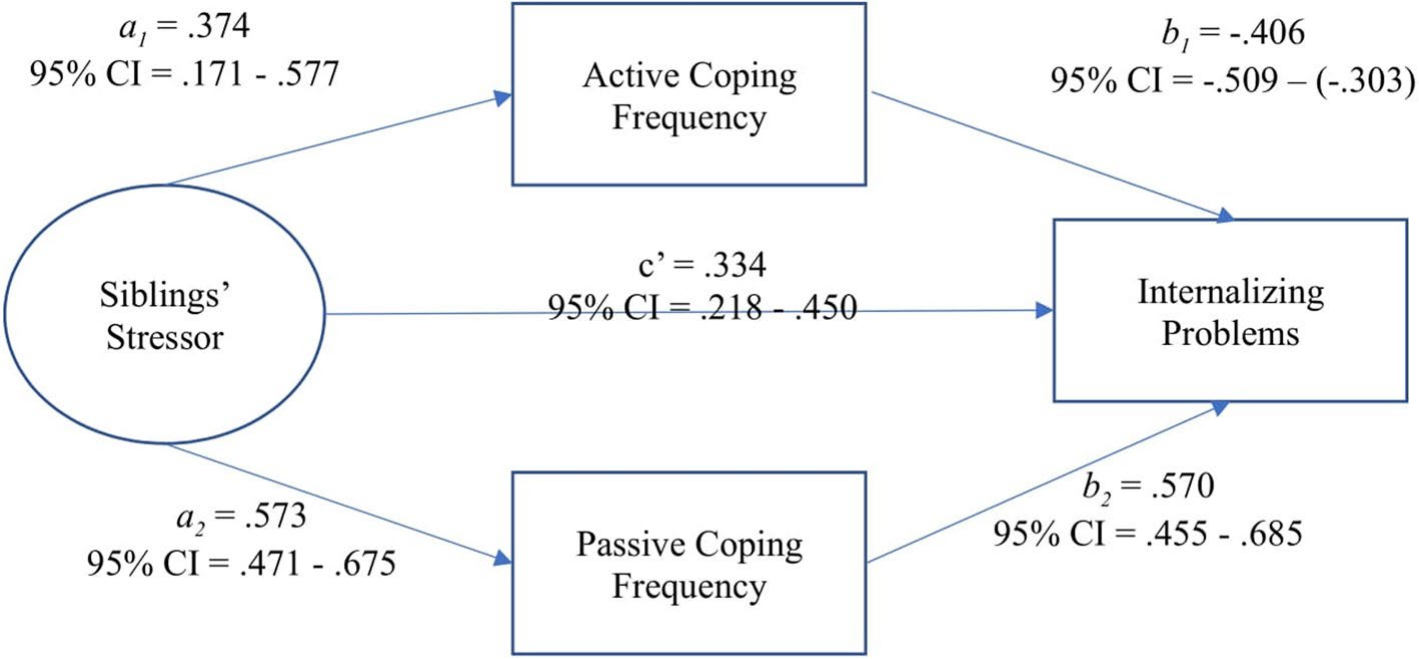
Mediation model for siblings’ internalizing problems. Note. Path coefficients are standardized. Only significant paths are reported. Covariates (time since first sought treatment, siblings’ age, and household income) and covariances were included in model estimation but are omitted from the figure for clarity. The diagram represents the pooled multilevel mediation model; subgroup-specific indirect effects by treatment duration are presented in Table 4 and described in the text

**Fig. 2:**
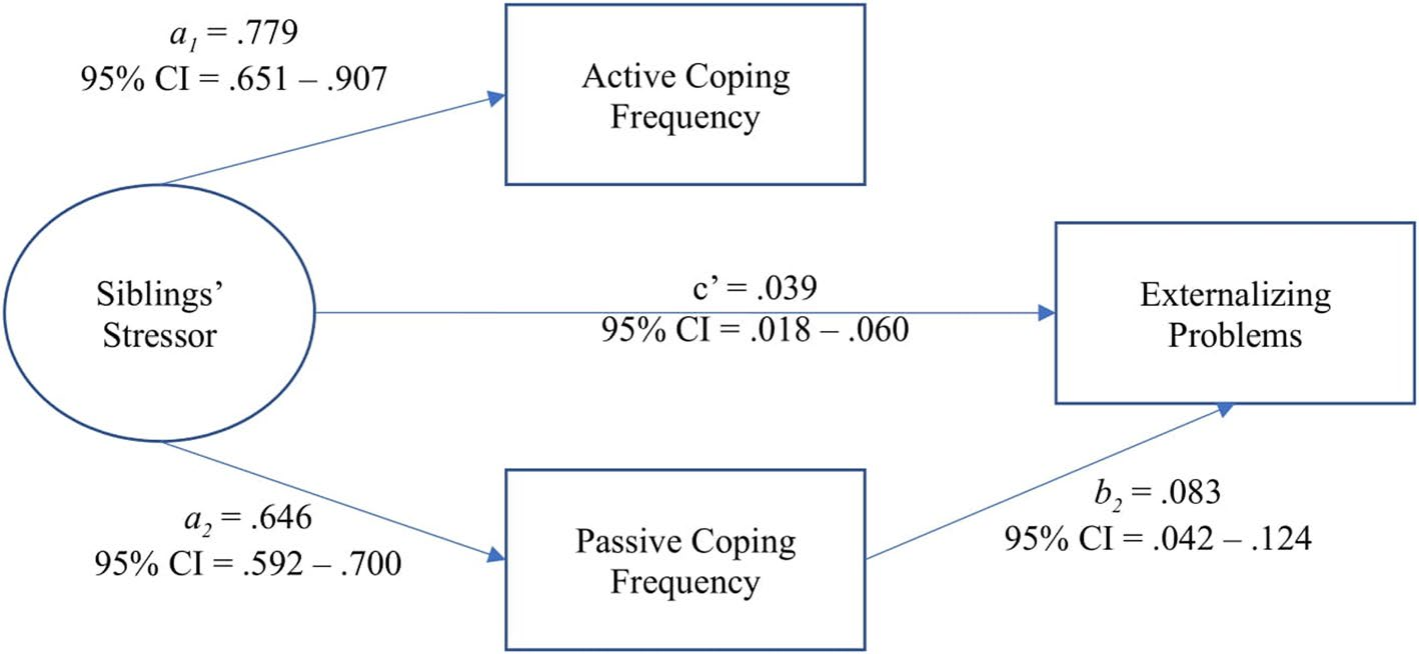
Mediation model for siblings’ externalizing problems. Note. Path coefficients are standardized. Only significant paths are reported. Covariates (time since first sought treatment, siblings’ age, and household income) and covariances were included in model estimation but are omitted from the figure for clarity. The diagram represents the pooled multilevel mediation model; subgroup-specific indirect effects by treatment duration are presented in Table 4 and described in the text

**Fig. 3:**
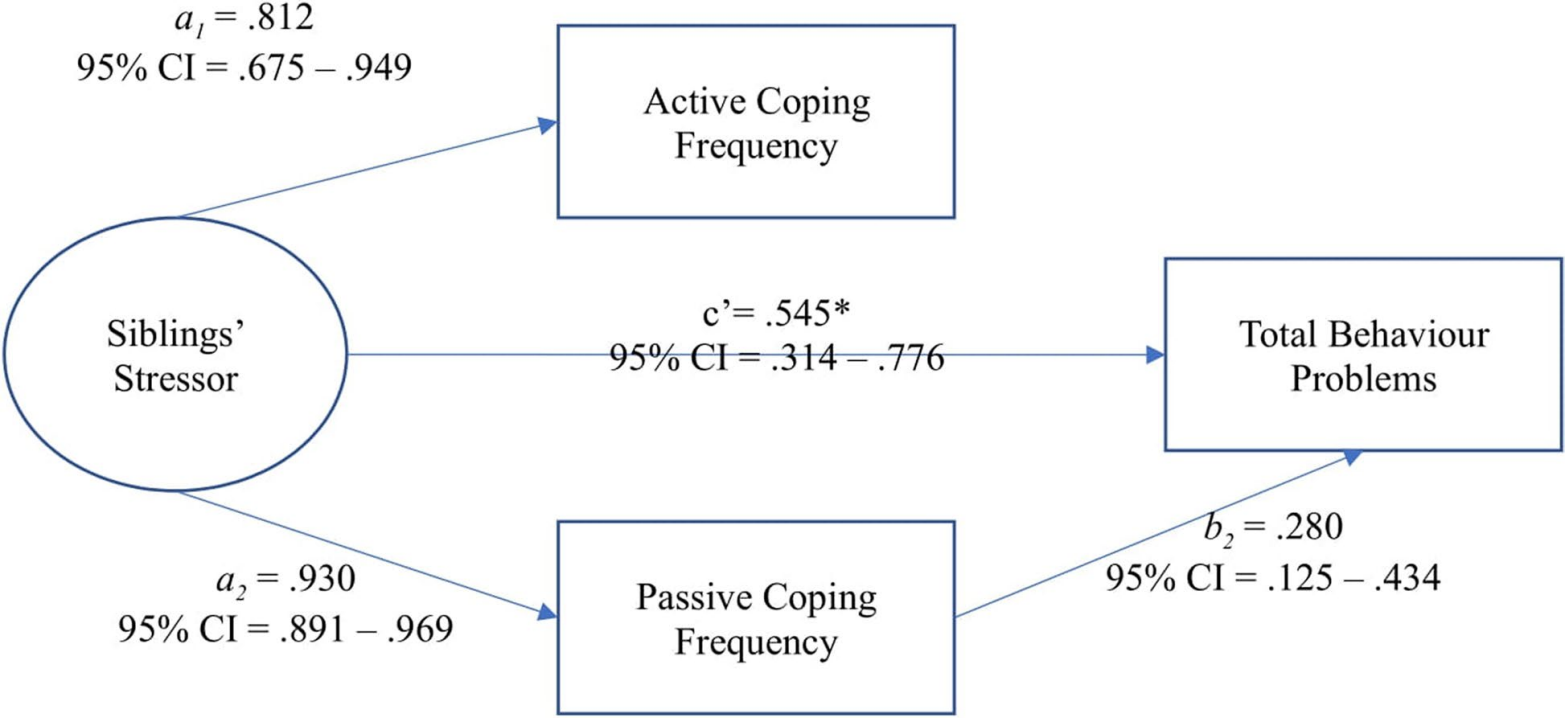
Mediation models for siblings’ total behaviour problems. Note. Path coefficients are standardized. Only significant paths are reported. Covariates (time since first sought treatment, siblings’ age, and household income) and covariances were included in model estimation but are omitted from the figure for clarity. The diagram represents the pooled multilevel mediation model; subgroup-specific indirect effects by treatment duration are presented in Table 4 and described in the text

The multilevel mediation analyses indicated variation in indirect effects by subgroup (≤ 1 year vs > 1 year; see Table 4), although the overall structural model remained consistent across groups (Figs. 1, 2 and 3). For internalizing problems (Fig. 1), both active and passive coping pathways showed significant mediation effects across treatment duration groups, with active coping demonstrating protective effects for both ≤ 1 year (−0.052, 95% CI [−0.103, −0.001]) and > 1 year groups (−0.109, 95% CI [−0.121, −0.097]), while passive coping showed risk effects (0.074, 95% CI [0.052, 0.096] and 0.068, 95% CI [0.041, 0.095], respectively). For externalizing problems (Fig. 2), active coping showed contrasting effects between duration groups, with protective effects in the ≤ 1 year group (−0.018, 95% CI [−0.029, −0.007]) but counterproductive effects in the > 1 year group (0.048, 95% CI [0.021, 0.075]), while passive coping was significant only in early treatment (0.054, 95% CI [0.021, 0.087]). Total behaviour problems (Fig. 3) exhibited the most pronounced between-group differences: active coping was associated with lower total problems in the ≤ 1 year group (−0.114, 95% CI [−0.185, −0.043]) and higher total problems in the > 1 year group (0.095, 95% CI [0.053, 0.136]), while passive coping was significant only in the ≤ 1 year group (0.149, 95% CI [0.003, 0.295]). These findings demonstrate that while passive coping primarily represents a risk pathway for families in early treatment stages, active coping shows differential associations with behavioural outcomes depending on treatment-duration group, with protective associations in families whose child has been in treatment for ≤ 1 year and positive associations with externalizing and total problems in families whose child has been in treatment for > 1 year.

**Table 4:** Mediated effects by time since first sought treatment subgroups

Mediated paths	Time since first sought treatment (≤ 1 year)	Time since first sought treatment (> 1 year)
Siblings’ Stressor → active coping → internalizing problems	-.052 (-.103 – [-.001]) *	-.109 (-.121 – [-.097]) *
Siblings’ Stressor → passive coping → internalizing problems	.074 (.052 –.096) *	.068 (.041 –.095) *
Siblings’ Stressor → active coping → externalizing problems	-.018 (-.029 – [-.007]) *	.048 (.021 –.075) *
Siblings’ Stressor → passive coping → externalizing problems	.054 (.021 –.087) *	.027 (0.01–0.044) *
Siblings’ Stressor → active coping → total behaviour problems	-.114 (-.185 – [-.043]) *	.095 (.053 –.136) *
Siblings’ Stressor → passive coping → total behaviour problems	.149 (.003 –.295) *	.078, (0.02–0.14)*

Only significant paths are reported and are noted with an *

## Discussion

This study examined how cumulative stress influences sibling adjustment in families with children with Q3 conditions. Multilevel mediation analyses revealed that parental psychological distress and caregiving burden were associated with all behavioural outcomes. Active coping was protective against internalizing problems across both treatment-duration groups, but showed contrasting patterns for externalizing and total behaviour problems: protective effects in the ≤ 1 year group versus positive associations in the > 1 year group. Passive coping was associated with negative outcomes, particularly for families in the ≤ 1 year treatment group. Time since first seeking treatment thus emerged as a critical contextual factor shaping how coping strategies relate to different behavioural domains. However, given the difference in group sizes (≤ 1 year: n = 12; > 1 year: n = 58), these between-group differences should be interpreted with considerable caution, as they may partly reflect sampling variability or model instability rather than substantively different processes across treatment-duration groups. More broadly, although data were collected across three time points, the mediation models focused on initial levels rather than change over time, which limits the study to cross-sectional inference. All mediation findings should therefore be interpreted as associational rather than causal.

Consistent with our first hypothesis, Siblings' Stressor demonstrated significant positive associations with all three behavioural outcomes: internalizing, externalizing, and total behaviour problems. These findings align with the contextual threat model [32] and provide empirical support for the cumulative impact of parental psychological distress and caregiving burden on sibling adjustment [4, 33]. The strength and consistency of these direct effects across all behavioural domains highlight the pervasive nature of stress transmission within families facing progressive, life-limiting conditions, supporting Sameroff's [45] emphasis on the combined influence of ecological risk factors. Our empirical derivation of the Siblings' Stressor construct revealed that parental psychological factors and caregiving burden were the most salient indicators of cumulative family stress within the variables available in this dataset [54]. However, it is important to acknowledge that the present study's assessment of broader family system variables was quite limited. While these findings suggest that parental distress and caregiving burden may constitute a core threat context for siblings, it is unknown if other contextual variables not assessed here, such as cultural identity or the family's social support network, play an equally or more significant role in sibling functioning. Future research should incorporate these broader domains to more fully operationalize Long et al.'s contextual threat model. As such, the derived construct is best understood as a partial operationalization of Long et al.'s broader framework, focused specifically on parental factors.

Our findings regarding active coping revealed complexity that challenges traditional assumptions about uniformly adaptive coping strategies [31]. While active coping was associated with fewer internalizing problems across both treatment-duration groups, its relationship with externalizing and total behaviour problems varied by treatment duration: protective in the ≤ 1 year group but positively associated with difficulties in the > 1 year group. Importantly, the active coping composite in this study encompassed several Kidcope strategies, including problem-solving, cognitive restructuring, and support seeking. Thus, our findings reflect the combined influence of multiple engagement-oriented strategies rather than problem-focused coping alone. This domain- and duration-specific pattern aligns with transactional perspectives emphasizing person–environment fit in coping effectiveness, whereby problem-focused strategies prove most beneficial when stressors are at least partly controllable but may become less effective under chronic, unmodifiable demands [6, 12, 31]. For example, cognitive restructuring and positive reframing may help siblings manage internal emotional distress even when the illness trajectory cannot be altered, whereas repeated problem-focused efforts in the context of largely uncontrollable demands may be less effective, though the mechanisms underlying this pattern require further investigation. Similarly, seeking support may be protective when family systems are responsive but less effective when caregiving demands constrain parental availability.

Prior work in pediatric chronic illness has similarly shown that active or primary-control coping relates more strongly to internalizing than externalizing symptoms, with adaptiveness depending on family climate and illness context [7, 13, 22–24]. Our results extend this literature by suggesting that, for siblings of children with Q3 conditions, active coping may remain effective for managing internal emotional distress, but may be less beneficial for outward behavioural responses in families further along a prolonged treatment trajectory. This highlights the need to identify which coping approaches remain supportive under prolonged caregiving demands.

These differential associations should also be considered in light of the progressive nature of Q3 conditions, including the fact that 14% of longer-treatment families experienced the child's death during the study. The longer-treatment group likely includes families navigating greater illness demands and, in some cases, bereavement, which may shape the context in which coping strategies operate. These are plausible contextual factors that could help explain the observed pattern, though the present data do not allow for direct examination of these mechanisms, and caution is warranted in interpreting group differences given the imbalanced sample sizes.

As hypothesized, passive coping was associated with higher levels of all behavioural outcomes, though the strength of these associations varied by treatment duration. Passive coping showed relatively similar associations with internalizing problems across both treatment-duration groups, indicating that avoidance, withdrawal, wishful thinking, and resignation link to greater emotional difficulties regardless of treatment duration. Although resignation may sometimes reflect realistic recognition of uncontrollable stressors, disengagement may limit opportunities for emotional processing and support-seeking, which may contribute to sustained distress. It is also worth noting that passive coping, as measured here encompasses strategies that may vary in how helpful or not they are in practice. While avoidance and withdrawal are broadly considered maladaptive, wishful thinking may serve a temporarily protective emotional function, and resignation in the context of an uncontrollable stressor may reflect realistic acceptance rather than helplessness [12].

However, associations between passive coping and externalizing and total behaviour problems were more pronounced in early-treatment families. This pattern indicates that while passive coping remains problematic throughout the illness trajectory, it relates most strongly to behavioural functioning during the initial treatment period when families first adapt to the diagnosis and treatment demands. During this early phase, disengagement strategies may limit emotional processing and available support, potentially contributing to frustration and behavioural difficulties as siblings navigate rapidly changing family dynamics. These findings align with research linking disengagement-oriented coping strategies to sustained anxiety, depression, and poorer behavioural adjustment in pediatric populations facing chronic illness-related stress [12, 23, 24]. These interpretations should nonetheless be made with caution, given the considerable conceptual overlap between passive coping, externalizing behaviour, and trauma symptomatology in children. For example, disengagement and withdrawal, features of the passive coping composite used here, can also present as dissociative responses in children exposed to chronic stress or trauma. Similarly, emotional dysregulation and oppositional behaviour, which comprise elements of the CBCL externalizing scale, are well-documented symptoms of childhood trauma [2]. Because trauma symptoms were not assessed in the present study, it is possible that the observed associations between passive coping and externalizing outcomes partially reflect shared variance with unassessed trauma responses rather than distinct coping and behavioural processes. Future studies examining sibling adjustment in this population may consider incorporating trauma symptom measures to disentangle these constructs and more precisely characterize the pathways linking family stress, coping, and behavioural outcomes.

These patterns point to meaningful domain specificity in how coping operates. Active coping's consistent protective effects on internalizing problems suggest that emotional regulation, problem-solving, and help-seeking may be relevant to the management of internal emotional states regardless of illness duration, whereas its links to externalizing and total behaviour problems are more context-dependent, shaped by the controllability of stressors and the resources available within the family system.

### Strengths and limitations

This study's strengths include its multi-site, prospective longitudinal design and the use of multilevel analyses that account for siblings' nested structure within families [25]. The empirical derivation of the Siblings' Stressor construct through multilevel factor analysis provides a data-driven approach to understanding family stress in this population. Additionally, examining both active and passive coping strategies with attention to treatment-duration differences allows for a nuanced understanding of adaptive versus maladaptive coping patterns across illness phases. Although data were collected over a decade ago, this dataset remains uniquely valuable given the absence of other longitudinal studies examining sibling adjustment in Q3 conditions. The treatment-duration patterns we identified may reflect psychosocial processes that remain relevant today. Although the policy and service landscape for families of children with life-limiting conditions has evolved since data collection (2009–2012), with advances in pediatric palliative care integration and growing awareness of sibling needs in some health systems, the core psychosocial processes examined here are unlikely to have changed fundamentally. Nevertheless, families today may have access to different levels of support than those in the CTT cohort, and this should be considered when generalizing the findings to current clinical contexts.

Several limitations warrant consideration. Our models focus on initial levels of all constructs and treat time since first seeking treatment as a between-family grouping variable, precluding conclusions about within-family change over time or the temporal ordering of stress, coping, and behavioural outcomes. Future research should employ longitudinal designs (e.g., cross-lagged panel or growth curve models) to examine how coping strategies and behavioural adjustment co-develop across the illness trajectory and to establish temporal precedence. The substantial direct effects of Siblings' Stressor on behavioural outcomes, even after accounting for coping, indicate that other important mediating mechanisms remain unexplored. Although multilevel analyses were used to account for the nested structure of siblings within families, the relatively small number of families contributing more than one sibling (6 families with two siblings and 4 with three) means that between-family (Level-2) estimates were derived from a limited number of clusters and should be interpreted with caution. This constraint is especially consequential for three aspects of the present analyses. First, the multilevel EFA was conducted with few family-level clusters, which risks overfitting and an unstable factor structure at Level-2; the resulting Siblings' Stressor construct, comprising only parental distress and caregiving burden, may therefore represent a partial operationalization of the broader contextual threat framework rather than a complete picture of the family stressors relevant to sibling adjustment. Second, the subgroup comparisons between the ≤ 1 year and > 1 year treatment-duration groups are based on a highly imbalanced split (n = 12 vs. n = 58), and observed differences may reflect sampling variability or model instability rather than true group differences. Third, mediation pathways estimated at both levels of the model are more uncertain at Level-2, given the limited clustering. These constraints mean this study is best understood as exploratory, and findings, particularly those involving between-family processes, subgroup differences, and the scope of the stressor construct, require replication with larger, more deeply clustered samples before conclusions can be drawn.

Several additional analytic constraints warrant acknowledgment. Despite the longitudinal design, mediation models focused on intercepts only, which limits the study to cross-sectional inference; temporal or developmental conclusions about how coping mediates stress over time cannot be drawn from these analyses. Relatedly, the indirect effects reported here reflect statistical associations rather than causal mediation and should not be interpreted as evidence that coping mechanisms causally impact stress on behavioural outcomes. Furthermore, several correlations in the present study approached an almost perfect linear relationship, most notably between Siblings' Stressor and internalizing problems (0.982), which raises concerns about potential construct overlap or measurement redundancy between the stressor composite and behavioural outcomes. These high correlations may have contributed to inflated path estimates, and the degree to which these constructs capture genuinely distinct phenomena requires further examination in future research.

Our Siblings' Stressor factor was designed to capture stressors specific to the ill child's condition and family caregiving context, but did not assess stressors in other key developmental settings, such as school, peer relationships, or the broader community, which likely interact with illness-related stress and coping to shape siblings' adjustment. We also did not assess parent–child relationship quality from the sibling's perspective. Given that relationship security, warmth, and conflict influence children's coping and mental health, the absence of a sibling-reported parent–child relationship measure limits our ability to examine how these relational processes shape or moderate the impact of contextual threat and coping on adjustment. Future studies informed by ecological and developmental systems perspectives should incorporate multi-context stress measures to better situate illness-related stress within siblings' broader lived environments.

Given the secondary design of this study, we were unable to administer the Kidcope in interview format for younger children; siblings aged 7–12 years completed the questionnaire independently. Without formal literacy assessment, reading or comprehension difficulties in some younger siblings may have introduced measurement error into coping variables, potentially attenuating or distorting associations with behavioural outcomes. We also could not examine precise age spacing between the ill child and siblings, nor the siblings’ age at the time of the ill child’s diagnosis – both of which likely shape caregiving roles, stress exposure, and the developmental context in which coping patterns were formed. Given the wide age range in our sample (7–18 years), siblings who were diagnosed at young age may have had different experiences compared to those for whom the illness began during adolescence; future work should assess these variables more directly.

Although gender and birth order were initially examined as potential covariates, neither showed a significant association with behavioural outcomes in this sample. However, given the modest sample size and limited statistical power, these null findings should not be interpreted as evidence that these variables are unimportant. Prior developmental research suggests that sibling gender and birth order may meaningfully shape caregiving roles, stress exposure, and coping processes [1, 18], and larger studies are needed to examine these effects. The passive coping composite aggregated strategies that may differ in how helpful or harmful they are in practice, and the present study was therefore unable to disentangle whether specific strategies within this grouping operated differently. Future research using item-level or subscale-level analyses would allow for a more nuanced examination of whether individual passive strategies carry distinct risks or benefits for sibling outcomes. 

We also did not account for whether participating siblings had another healthy sibling in the household, which may shape their experience of illness-related stress and coping. A healthy sibling companion could provide social support and a buffer against isolation, whereas being the sole healthy sibling may intensify caregiving expectations and reduce opportunities for peer-level support within the family. To our knowledge, this variable has not been systematically examined in the literature on siblings of children with life-limiting conditions, and future studies should consider sibling composition as a potentially meaningful contextual factor. We did not differentiate maternal and paternal reports in our models. Because only one designated primary caregiving parent per family completed questionnaires, and the large majority of respondents were mothers (*n* = 54) with only four fathers, we focused on a single family-level indicator of contextual threat. Although the four father respondents represent approximately 7% of the sample, we were unable to conduct a formal sensitivity analysis excluding father respondents, which is a limitation of the present study. Future research with prospective designs and clearly separated maternal and paternal data would allow for sensitivity testing of parent gender effects and examination of potential parent-specific pathways. We were also unable to model illness stage or bereavement status explicitly; given that a subset of children died during the assessment period, siblings' adjustment trajectories were likely shaped by proximity to the end of life and bereavement, which future longitudinal studies should examine in more detail.

The counterintuitive pattern in which active coping associates differently with externalizing and total behaviour problems across treatment durations requires replication and mechanistic investigation. Additionally, our dichotomous treatment duration variable (≤ 1 year vs. > 1 year) may not capture the full complexity of how stress and coping relate to behavioural outcomes across illness phases, and future studies should consider more fine-grained representations of duration and illness stage.

### Practice and research implications

These findings highlight the importance of time-sensitive interventions, as siblings in families receiving treatment for less than one year were more likely to rely on passive coping. However, this pattern is preliminary and should be interpreted with caution, given the very small number of siblings in the ≤ 1 year group (*n* = 12). While our findings suggest that the first year of treatment may represent an important period for coping intervention, replication with a larger sample is needed before drawing firm conclusions about a critical early window for prevention [35]. These findings suggest that programs could consider prioritizing early interventions that strengthen active coping and prevent reliance on passive strategies. As treatment progresses, sibling coping needs may change. Interventions may need to help siblings notice when continued efforts to change or control largely uncontrollable illness-related stressors become exhausting or less helpful, and to introduce approaches that encourage acceptance, emotional regulation, and flexible disengagement from unchangeable aspects of the situation. Parental psychological distress emerged as a central source of family-level stress, underscoring that interventions targeting parental mental health and caregiving demands may yield downstream benefits for healthy siblings. Family-centered approaches that address both parental mental health and sibling coping are therefore essential across all treatment phases, and providers should implement regular screening for sibling adjustment that accounts for the treatment timeline, coping patterns, and domain-specific symptoms. Even siblings with strong coping skills may still require support when family stress levels remain high.

Future research could consider investigating why the association between active coping and behavioural outcomes differs across treatment-duration groups, including whether particular components of active coping, such as problem-focused efforts versus support seeking, function differently at different points in the illness trajectory. This includes examining potential mediators such as coping efficacy, resource depletion, family adaptation, and environmental change (Compas et al., 2017; Zimmer-Gembeck & Skinner, 2016), and determining whether the patterns observed reflect overuse of particular strategies, misapplication of active coping to uncontrollable stressors, or broader changes in family dynamics and resources over time.

To guide intervention design, researchers should identify optimal time points for adapting support, test early preventive approaches, and consider using cross-lagged panel designs to explore bidirectional relationships between stressors, coping, and outcomes. Residual effects may be further explained by contextual factors not captured in the present study, including family communication, sibling-specific social support, peer and school environments, and healthcare system influences. Future studies should examine whether time-adapted interventions improve outcomes and whether early prevention can shape long-term adjustment in siblings of children with life-limiting conditions.

## Conclusion

This study demonstrates that healthy siblings of children with progressive life-limiting conditions face significant psychological challenges associated with parental distress and caregiving demands. Coping strategies showed differential associations with behavioural outcomes, with active coping linked to fewer internalizing difficulties and passive coping associated with greater behavioural problems. Patterns tentatively varied by treatment-duration group, though these differences should be interpreted cautiously given the exploratory nature of the analyses and the small sample in the shorter-treatment group. These findings offer a preliminary contribution to understanding sibling adjustment in the context of life-limiting illness and point toward the value of early, family-centred approaches that account for coping patterns and illness phase. Replication with larger samples is needed to establish the robustness of these associations and inform intervention design.

## Supplementary Information

Supplementary Material 1.

Supplementary Material 2.

## Data Availability

No datasets were generated or analysed during the current study.
