# Supplementary material for: Coping as a mediator between family stressors and behavioural adjustment in siblings of children with progressive life-limiting conditions: a multilevel longitudinal study
Source: BMC Pediatr. 2026 Jun 8;26:751. doi: 10.1186/s12887-026-07110-z (PMC13474505; doi:10.1186/s12887-026-07110-z)
Supplement: Supplementary file 1 [file 12887_2026_7110_MOESM1_ESM.docx]

**Supplementary Table S1.**

*Standardized factor loadings for the Siblings’ Stressor latent variable across three time points*

| **Indicator** | **Time 1 loading** | **Time 2 loading** | **Time 3 loading** |
| --- | --- | --- | --- |
| Parental stress | .852 | .937 | .934 |
| Parental anxiety | .854 | .842 | .961 |
| Parental depression | .842 | .860 | .897 |
| Parental burden of caregiving | .602 | .857 | .745 |

*Note.* Only indicators with strong and consistent loadings across time (parental stress, anxiety, depression, and burden of caregiving) were retained in the final Siblings’ Stressor factor; other candidate variables (e.g., family cohesion, marital satisfaction, grief, symptom severity) did not meet the loading criteria of >.40 and were excluded from the final model.
